# Associations between fish consumption and metabolic syndrome. A large cross-sectional study from the Norwegian Tromsø Study: Tromsø 4

**DOI:** 10.1186/s13098-016-0137-5

**Published:** 2016-03-03

**Authors:** C. Tørris, M. Molin, M. Cvancarova Småstuen

**Affiliations:** Oslo and Akershus University College, Oslo, Norway; Bjorknes University College, Oslo, Norway; Institute of Basic Medical Sciences, University of Oslo, Blindern, Oslo, Norway

**Keywords:** Metabolic syndrome, Insulin resistance, Diet, Fish consumption, Fatty fish, Lean fish

## Abstract

**Background:**

Fish consumption may prevent or improve metabolic health. The aim of this study was to identify associations between fish consumption, both fatty and lean, and metabolic syndrome and its components.

**Methods:**

Associations between fish consumption and metabolic syndrome and its components were studied in a large sample from a Norwegian population (N = 23,907), using cross-sectional data from the Tromsø 4 survey (1994–1995). Metabolic syndrome was defined using the JIS definition, and dietary data was collected using food frequency questionnaires (FFQ). Blood samples were taken for biochemical assessments, and anthropometric and blood pressure measurements were carried out according to standard protocols.

**Results:**

In this sample from an adult population (aged 26–70 years, mean age 44 years, SD 11.69, 48 % men), a higher fish consumption (≥1/week) was associated with a healthier lipid profile with increased HDL-C and decreased TG. Participants aged 60–70 years consuming fish once a week or more had significantly lower risk of having MetS, compared to those consuming fish less than once a week (OR 0.64, CI 0.45–0.91). When investigating fatty and lean fish separately, only lean fish consumption was associated with a reduced the risk of having MetS. Participants aged 60–70 years consuming lean fish once a week or more, had lower risk of having MetS compared to those consuming lean fish less than once a week (OR 0.65, CI 0.48–0.87). No association was found for consumption of fatty fish, or for lean fish in the age groups <45 or 45–59 years.

**Conclusions:**

These results indicates that fatty and lean fish consumption influences MetS risk differently, possibly also related to age. However, further investigation is needed to establish how various fish consumption may influence MetS and its components, particularly when stratified by fatty and lean fish.

## Background

Metabolic syndrome (MetS) is a cluster of risk factors for cardiovascular disease (CVD) and diabetes mellitus type 2 (DM2) with metabolic abnormalities including abdominal obesity, dyslipidaemia, hyperglycaemia, and hypertension [1, 2]. Several definitions and diagnostic criteria for MetS have been proposed, the latest by the new joint interim societies (JIS) [2]. MetS has been associated with a doubling of CVD risk as well as a fivefold increased risk of DM2 [3]. The syndrome affects public health, and the increased risk of morbidity and mortality is profound [4].

Lifestyle interventions based on general recommendations may reduce the metabolic abnormalities, however few studies have investigated associations between fish consumption and MetS prevalence [5]. One follow-up study [6] and three cross-sectional studies [7–9], found associations between fish consumption and MetS. However, only one study found associations among women [9]. Despite limited documentation, the results suggest that fish consumption may have a preventive role in the development of MetS and possibly improve metabolic health. Moreover, the above association might vary by gender [5].

Therefore, the aim of this study was to identify associations between total, fatty and lean fish consumption and MetS and its components, in a large sample from an adult population from Northern Norway. Our overall hypothesis is that higher fish consumption is associated with a healthier metabolic profile and a lower prevalence of MetS, and that there might be age and gender differences in the prevalence of MetS.

## Methods

### Study design, settings and participants

This cross-sectional study is based on data from the fourth survey of the Tromsø Study (Tromsø 4). Tromsø 4 was conducted in 1994–95, and residents of Tromsø ≥25 years of age were invited to participate (N = 37,558). A total of 27,158 participants attended (attendance rate 77 %), and measurements were carried out according to standard protocols [10]. Both study design and population have been described previously [10]. The Tromsø study is a longitudinal population-based health study carried out in Northern Norway, consisting of several repeated surveys. The first survey was initiated in 1974 (Tromsø 1), due to the high mortality of cardiovascular diseases [10]. A large representative sample of the Tromsø population, based on the official population registry, was invited to participate. Repeated measurements were taken, where the subjects were free to attend whenever suitable within the time frame of each survey (1 year). Non-attendees were given one reminder. In these random sampled birth cohorts the surveys followed the same design [10], where each survey was conducted in two phases with basic examination in the first visit and more extensive examinations in the second visit 2–4 weeks after the first visit [10]. Both earlier participants as well as a random sample were invited to the surveys. Data collected consisted of questionnaires and measurements. The Tromsø study is funded by the University of Tromsø, the National Screening Services, the Research Council of Norway, Northern Norway Regional Health Authority, Norwegian Council on Cardiovascular Diseases and Norwegian Foundation for Health and Rehabilitation [10].

### Questionnaires and serum samples

In the invitation to participate, a questionnaire was enclosed in the invitation letter. In the first visit blood pressure and blood sample for serum measurements was taken. After the first visit participants were given a second questionnaire consisting of questions regarding dietary habits, dietary supplements and pregnancy (parity, lactation length in months per child) to return by mail after the first visit. Fish consumption was assessed in weekly frequencies in the questionnaire (never, <1, 1, 2–3, 4–5, approximately every day), including consumption of fatty fish such as salmon and lean fish such as cod. Nutrients were computed and are described elsewhere [11]. In the second visit anthropometrical measurements and further non-fasting serum samples such as plasma glucose were taken.

### Metabolic syndrome

MetS was defined using the JIS definition [2], where a presence of any three of five given risk factors constitutes a diagnosis of MetS. For central obesity the IDF cut points were used (WC ≥94 cm in men and ≥80 cm in women) [12]. For the other components, the cut points were as follows: S-triglycerides ≥150 mg/dL (1.7 mmol/L), S-HDL cholesterol <40 mg/dL (1.0 mmol/L) in men and <50 mg/dL (1.3 mmol/L) in women, S-glucose ≥100 mg/dL (5.5 mmol/L), systolic blood pressure ≥130 mm Hg and/or diastolic blood pressure ≥85 mm Hg. The plasma glucose measured in Tromsø 4 was not fasting.

### Statistical analyses

Data are presented stratified by gender and age-groups, due to hormonal differences in men and women, and changes in the body over a lifespan (pre- and postmenopausal women, aging). Age groups were defined as <45, 45–59, 60–70 years. Fish consumption was analysed as a categorical variable both as less/higher than once a week, and as never, <1, 1, 2–3 or more per week. MetS components were analysed both as continuous variables, and as categorical variables. To investigate crude associations between fish consumption and MetS prevalence or its components, Chi Square tests for categorical variables and analysis of variance (ANOVA) for continuous variables were used. Correlations between pairs of continuous variables were examined by Pearson’s correlation. Linear regression models were fitted to examine relationship between components of MetS (continuous) as dependent variable, and fish consumption (categorical) as independent variable. Logistic regression models were used for categorical variables, and potential confounding variables were adjusted for (gender, education, physical activity, single/living with a spouse, parity, lactation). P value < 0.05 was considered statistical significant. All tests were two-sided, and all CI is 95 % CI. Analyses were performed using IBM SPSS Statistics 22.

## Results

In the present study participants <70 years are included, and the study therefore consists of 23,907 participants, aged 26–70 years (mean age 44 years, SD 11.69), 48 % being men. Almost one-third of the participants reported consuming fatty fish at least once a week (31 %), whereas 77 % reported consuming lean fish and 81 % reported consuming fish for dinner at least once a week. Women in the age groups <45, 45–59, 60–70 years had given birth to 1.5, 2.4 and 2.9 child, and the average total lactation length was 14.5, 12.6 and 16.2 months, respectively.

### Fish consumption and components of metabolic syndrome

#### Associations revealed in the whole sample and among men and women individually

Using analysis of variance we investigated associations between fish consumption (fatty and lean fish) as a categorical variable (less than once a week/once a week or more), and the components of MetS (WC, S-TG, S-HDL-C, SBP, DBP, S-glucose) as continuous variables.

In the whole sample, those consuming fish once a week or more had a significantly higher HDL-C, SBP, DBP, and S-glucose. A higher borderline significant WC was observed in those consuming fish once a week or more in the whole sample (P = 0.06). Moreover, participants consuming fish once a week or more were also significantly older (P < 0.0001), and had a significantly higher energy intake than their counterparts (P < 0.0001) (Table 1).

**Table 1:** Characteristics of participants by consumption of fish, in total sample and by gender, mean (SD)

	Fish consumption		P^a^
	<1/week	≥1/week	
Age (years)			
Total	38.0 (9.66)	45.6 (11.59)	<0.0001
Women	38.3 (9.93)	45.0 (11.69)	<0.0001
Men	37.7 (9.37)	46.3 (11.44)	<0.0001
Waist circumference (cm)			
Total	88.6 (12.06)	89.7 (11.27)	0.06
Women	83.4 (11.17)	84.0 (10.72)	0.5
Men	94.2 (10.40)	95.0 (9.00)	0.3
Triglycerides (mmol/l)^c^			
Total	1.52 (1.05)	1.51 (1.03)	0.7
Women	1.22 (0.77)	1.28 (0.82)	0.006
Men	1.83 (1.20)	1.77 (1.18)	0.04
HDL-cholesterol (mmol/L)^b, c^			
Total	1.45 (0.38)	1.51 (0.41)	<0.0001
Women	1.60 (0.38)	1.65 (0.40)	<0.0001
Men	1.28 (0.31)	1.35 (0.35)	<0.0001
Systolic blood pressure (mm hg)			
Total	132.1 (15.8)	135.6 (18.3)	<0.0001
Women	127.3 (15.90)	131.8 (18.78)	<0.0001
Men	137.2 (14.06)	139.8 (16.75)	<0.0001
Diastolic blood pressure (mm hg)			
Total	77.2 (11.7)	79.8 (12.8)	<0.0001
Women	75.0 (11.53)	77.5 (12.78)	<0.0001
Men	79.5 (11.46)	82.3 (12.41)	<0.0001
Glucose (mmol/L)^c^			
Total	4.76 (1.18)	4.87 (1.24)	0.05
Women	4.80 (1.30)	4.81 (1.11)	0.9
Men	4.69 (0.97)	4.95 (1.38)	0.01
Energy intake (MJ/day)			
Total	7.51 (2.16)	8.07 (2.21)	<0.0001
Women	6.41 (1.59)	6.85 (1.61)	<0.0001
Men	8.66 (2.08)	9.39 (2.00)	<0.0001

Consumption of fish: less than once a week/once a week or more. Numbers of participants vary because of missing information variables

The Tromsø Study: Tromsø 4

^a^ P value by one-way analysis of variance

^b^ *HDL-cholesterol* high-density lipoprotein cholesterol

^c^ Plasma glucose are non-fasting

Interestingly, an opposite association for TG among men and women were found when investigating associations between fish consumption and the components of MetS among men and women individually. Fish consumption once a week or more was significantly associated with higher TG in women and a lower TG in men. When the whole sample was analysed the above listed association disappeared (Table 1).

For both genders, fish consumption once a week or more was associated with a statistically significantly increased HDL-C as well as an increased blood pressure. Surprisingly, also an increased S-glucose among men consuming fish once a week or more was found, compared to those consuming fish less than once a week (Table 1).

### Consumption of fatty and lean fish, and components of MetS

In linear regression models we investigated associations between consumption of both fatty and lean fish (never, <1, 1, 2–3 or more per week) respectively as independent variables and the different components of MetS (WC, S-TG, S-HDL-C, SBP, DBP, S-glucose) as dependent variables both in the whole sample, and genderwise (Table 2).

**Table 2:** Estimated change in components of metabolic syndrome by increasing consumption of fatty and lean fish

	Total sample			Women			Men		
	B	95 % CI	P value	B	95 % CI	P value	B	95 % CI	P value
Fatty fish									
WC									
Unadjusted	0.981	0.564 to 1.397	<0.0001	0.870	0.283 to 1.458	0.004	0.737	0.281 to 1.192	0.002
Age adjusted	0.580	0.160 to 0.999	0.007	0.219	−0.366 to 0.804	0.5	0.472	0.013 to 0.931	0.04
Multivariable adjusted^a^	0.417	−0.087 to 0.920	0.1	0.774	0.046 to 1.502	0.04	0.707	0.164 to 1.250	0.01
S-TG									
Unadjusted	0.022	0.003 to 0.040	0.02	0.030	0.010 to 0.051	0.004	−0.020	−0.050 to 0.010	0.2
Age adjusted	−0.011	−0.030 to 0.008	0.2	−0.021	−0.042 to 0.000	0.05	−0.029	−0.060 to 0.001	0.06
Multivariable adjusted^a^	−0.043	−0.064 to −0.022	<0.0001	−0.027	−0.050 to −0.004	0.02	−0.025	−0.060 to 0.010	0.2
HDL-C									
Unadjusted	0.021	0.014 to 0.028	<0.0001	0.027	0.017 to 0.037	<0.0001	0.035	0.026 to 0.043	<0.0001
Age adjusted	0.007	0.000 to 0.015	0.06	0.013	0.002 to 0.023	0.02	0.018	0.010 to 0.027	<0.0001
Multivariable adjusted^a^	0.025	0.017 to 0.033	<0.0001	0.012	0.000 to 0.024	0.05	0.017	0.007 to 0.027	0.001
SBP									
Unadjusted	2.379	2.063 to 2.694	<0.0001	2.812	2.356 to 3.269	<0.0001	1.417	1.009 to 1.826	<0.0001
Age adjusted	0.190	−0.115 to 0.495	0.2	−0.136	−0.559 to 0.287	0.5	0.073	−0.334 to 0.481	0.7
Multivariable adjusted^a^	−0.362	−0.698 to −0.025	0.04	−0.167	−0.637 to 0.303	0.5	−0.039	−0.493 to 0.416	0.9
DBP									
Unadjusted	1.649	1.424 to 1.875	<0.0001	1.586	1.269 to 1.904	<0.0001	1.412	1.103 to 1.720	<0.0001
Age adjusted	0.051	−0.166 to 0.268	0.6	−0.110	−0.416 to 0.196	0.5	−0.023	−0.319 to 0.274	0.9
Multivariable adjusted^a^	−0.196	−0.439 to 0.046	0.1	−0.013	−0-355 to 0.329	0.9	−0.073	−0.4008 to 0.262	0.7
Glucose^b^									
Unadjusted	0.050	0.008 to 0.092	0.02	−0.008	−0.060 to 0.044	0.8	0.113	0.044 to 0.182	0.001
Age adjusted	0.020	−0.022 to 0.063	0.3	−0.030	−0.083 to 0.023	0.3	0.078	0.009 to 0.148	0.03
Multivariable adjusted^a^	0.022	−0.028 to 0.071	0.4	−0.023	−0.080 to 0.034	0.4	0.088	0.004 to 0.171	0.04
Lean fish									
WC									
Unadjusted	0.687	0.279 to 1.095	0.001	0.394	−0.153 to 0.941	0.2	0.579	0.114 to 1.044	0.02
Age adjusted	0.026	−0.396 to 0.448	0.9	−0.472	−1.022 to 0.078	0.09	0.045	−0.445 to 0.534	0.9
Multivariable adjusted^a^	−0.713	−1.234 to −0.192	0.007	−0.450	−1.157 to 0.258	0.2	0.069	−0.524 to 0.663	0.8
S-TG									
Unadjusted	0.007	−0.010 to 0.024	0.4	0.043	0.024 to 0.062	<0.0001	−0.035	−0.063 to −0.008	0.01
Age adjusted	−0.037	−0.055 to −0.019	<0.0001	−0.016	−0.035 to 0.004	0.1	−0.052	−0.081 to −0.022	0.001
Multivariable adjusted^a^	−0.062	−0.082 to −0.043	<0.0001	−0.014	−0.035 to 0.007	0.2	−0.041	−0.075 to −0.008	0.02
HDL-C									
Unadjusted	0.028	0.022 to 0.035	<0.0001	0.023	0.013 to 0.032	<0.0001	0.038	0.030 to 0.046	<0.0001
Age adjusted	0.012	0.005 to 0.019	0.001	0.007	−0.003 to 0.017	0.2	0.016	0.007 to 0.024	<0.0001
Multivariable adjusted^a^	0.030	0.022 to 0.037	<0.0001	0.005	−0.006 to 0.016	0.4	0.012	0.003 to 0.022	0.01
SBP									
Unadjusted	2.731	2.438 to 3.024	<0.0001	3.328	2.907 to 3.749	<0.0001	2.014	1.632 to 2.395	<0.0001
Age adjusted	−0.034	−0.324 to 0.256	0.8	0.074	−0.322 to 0.470	0.7	0.081	−0.315 to 0.477	0.7
Multivariable adjusted^a^	−0.375	−0.690 to −0.060	0.02	0.393	−0.041 to 0.026	0.08	0.072	−0.364 to 0.508	0.7
DBP									
Unadjusted	1.776	1.568 to 1.984	<0.0001	1.655	1.364 to 1.946	<0.0001	1.837	1.551 to 2.124	<0.0001
Age adjusted	−0.227	−0.433 to −0.022	0.03	−0.214	−0.498 to 0.069	0.1	−0.214	−0.502 to 0.073	0.1
Multivariable adjusted^a^	−0.396	−0.596 to −0.142	0.001	−0.057	−0.373 to 0.260	0.7	−0.072	−0.393 to 0.249	0.7
Glucose^b^									
Unadjusted	0.063	0.022 to 0.104	0.003	0.011	−0.039 to 0.060	0.7	0.127	0.058 to 0.197	<0.0001
Age adjusted	0.021	−0.022 to 0.063	0.3	−0.015	−0.066 to 0.036	0.6	0.062	−0.011 to 0.136	0.1
Multivariable adjusted^a^	0.044	−0.006 to 0.094	0.09	0.033	−0.021 to 0.088	0.2	0.071	−0.021 to 0.163	0.1

Estimated change (regression coefficient B and 95 % confidence interval) in various components of metabolic syndrome (WC, TG, HDL, blood pressure, S-glucose) as the dependent variable, by an increasing consumption of fatty fish and lean fish, respectively (never, <1, 1, 2–3 or more per week). The Tromsø Study: Tromsø 4

*WC* waist circumference, *S-TG* S-triglycerides, *HDL-C* high density lipoprotein-cholesterol, *SBP* systolic blood pressure, *DBP* diastolic blood pressure

^a^ *Multivariable adjusted* age, energy intake

^b^ Plasma glucose are non-fasting

An increased consumption of both fatty and lean fish was associated with a higher WC for the total sample (unadjusted). However, in the multivariable model adjusting for age and energy intake a higher consumption of lean fish was associated with decreased WC (P = 0.007). Using the same model, an increased consumption of fatty fish was in contrast associated with a significant increased WC, for both men and women (Table 2).

An increased consumption of fatty fish was associated with a higher TG, both in the whole sample and among women. However, in the multivariable model adjusting for age and energy intake consumption of fatty fish was associated with a decreased TG both in the whole sample and among women. Among men no association between fatty fish and TG was found in the multivariable model. Interestingly, a higher consumption of lean fish was associated with a higher TG in women and lower TG in men. However, when adjusting for age and energy intake the association disappeared among women, but remained among men (P = 0.02) (Table 2).

Both an increased consumption of fatty fish and lean fish was associated with higher HDL-C, but the association did not remain statistically significant when adjusted for age and energy intake for women (Table 2).

Both an increased consumption of fatty fish and lean fish was associated with a slightly higher blood pressure. When adjusting for age and energy intake, consumption of both fatty and lean fish was associated with a slightly lower blood pressure in the whole sample, this association was however not statistically significant for consumption of fatty fish and DBP (Table 2).

An increasing consumption of both fatty and lean fish was associated with higher levels of glucose (non-fasting), both in the whole sample and among men. When adjusting for age and energy intake the association only remained significant for consumption of fatty fish among men and for consumption of lean fish in the whole sample (Table 2).

### Fish consumption and metabolic syndrome

In total, the prevalence of MetS was 8.1 % in the whole sample, with the estimates increasing with age (Table 3). In the whole sample, the prevalence of MetS was higher (8.8 %) among those consuming fish once a week or more, compared to those consuming fish less than once a week (4.8 %). Women who had given birth had a higher risk of having MetS (OR 2.69, CI 2.03–3.57), compared to those who had not given birth. Only 0.3 % was diagnosed with all five component of MetS, 1.6 % with four components, 6.2 % with three components, 17.1 % with two components, 31.9 % with one component and 42.9 % did not fulfil the criteria for any of the five component of MetS.

**Table 3:** Prevalence of metabolic syndrome (%) in different age groups

	N	%
Total sample	1927	8.1
Women	869	7.0
<45 years	107	1.6
45–59 years	342	8.6
60–70 years	420	25.8
Men	1058	9.2
<45 years	210	3.5
45–59 years	483	12.1
60–70 years	365	24.4

Metabolic syndrome criteria by the JIS definition: waist circumference ≥94 cm in men and ≥80 cm in women, triglycerides ≥150 mg/dL (1.7 mmol/L), HDL cholesterol <40 mg/dL (1.0 mmol/L) in men and <50 mg/dL (1.3 mmol/L) in women, glucose ≥100 mg/dL (5.5 mmol/L), systolic blood pressure ≥130 mm Hg and diastolic blood pressure ≥85 mm Hg. Plasma glucose was not fasting

The Tromsø Study: Tromsø 4

Fish consumption and associations with MetS as an entity was investigated among the different age groups, using logistic regression. Frequency of fish consumption was investigated both in total and as fatty and lean fish individually. In model 1 we adjusted for gender, in model 2 we further adjusted for education, physical activity, single/living with a spouse, and in model 3 we further adjusted for parity and lactation.

When investigating consumption of fatty and lean fish together after adjusting for gender (Model 1), participants aged 60–70 years consuming fish once a week or more, had 36 % lower risk of having MetS compared to those consuming fish less than once a week (OR 0.64, CI 0.45–0.91). When further adjusting for education, physical activity, single/living with a spouse (Model 2), the association remained statistically significant in the age group 60–70 years (P = 0.02) (Table 4).

**Table 4:** Fish consumption and associations with metabolic syndrome

Model	Age group	B	OR	95 % CI	P
Both fatty and lean					
1	<45 years	0.082	1.09	0.83–1.42	0.6
1	45–59 years	0.189	1.21	0.93–1.57	0.2
1	60–70 years	−0.444	0.64	0.45–0.91	0.01
2	<45 years	0.057	1.06	0.81–1.39	0.7
2	45–59 years	0.202	1.22	0.94–1.60	0.1
2	60–70 years	−0.427	0.65	0.46–0.93	0.02
3	<45 years	−0.142	0.87	0.52–1.45	0.6
3	45–59 years	0.104	1.11	0.72–1.70	0.6
3	60–70 years	−0.141	0.87	0.51–1.49	0.6
Fatty fish					
1	<45 years	0.207	1.23	0.95–1.59	0.1
1	45–59 years	0.124	1.13	0.97–1.33	0.1
1	60–70 years	0.126	1.13	0.94–1.37	0.2
2	<45 years	0.210	1.23	0.95–1.60	0.1
2	45–59 years	0.120	1.13	0.96–1.33	0.1
2	60–70 years	0.122	1.13	0.93–1.37	0.2
3	<45 years	0.324	1.38	0.87–2.20	0.2
3	45–59 years	0.179	1.20	0.91–1.57	0.2
3	60–70 years	0.211	1.24	0.91–1.67	0.2
Lean fish					
1	<45 years	0.023	1.02	0.79–1.32	0.9
1	45–59 years	0.043	1.04	0.84–1.30	0.7
1	60–70 years	−0.439	0.65	0.48–0.87	0.004
2	<45 years	0.000	1.00	0.77–1.30	1.0
2	45–59 years	0.066	1.07	0.85–1.34	0.6
2	60–70 years	−0.439	0.65	0.48–0.87	0.004
3	<45 years	−0.111	0.90	0.55–1.46	0.7
3	45–59 years	−0.045	0.96	0.66–1.38	0.8
3	60–70 years	−0.268	0.77	0.49–1.20	0.2

OR and P value by logistic regression (binary) with metabolic syndrome as dependent and frequency of fish consumption as independent (less than once a week/once a week or more)

Metabolic syndrome criteria by the JIS definition: waist circumference ≥94 cm in men and ≥80 cm in women, triglycerides ≥150 mg/dL (1.7 mmol/L), HDL cholesterol <40 mg/dL (1.0 mmol/L) in men and <50 mg/dL (1.3 mmol/L) in women, glucose ≥100 mg/dL (5.5 mmol/L), systolic blood pressure ≥130 mm Hg and diastolic blood pressure ≥85 mm Hg. Plasma glucose measured in Tromsø 4 was not fasting. Fish consumption (less than once a week/once a week or more). Numbers of participants vary because of missing information variables. The Tromsø Study: Tromsø 4

*Model 1* adjusted for gender

*Model 2* further adjusted for education, physical activity, living with a spouse

*Model 3* further adjusted for parity, lactation

When investigating fatty and lean fish separately we found that consumption of lean fish was associated with decreased risk of having MetS. Participants aged 60–70 years consuming lean fish once a week or more, had 35 % lower risk of having MetS compared to those consuming lean fish less than once a week (OR 0.65, CI 0.48–0.87). When further adjusting for education, physical activity, single/living with a spouse (Model 2), the association remained significant in the age group 60-70 years (OR 0.65, CI 0.48–0.87) (Table 4).

However, these associations reached the level of statistical significance only for the participants aged 60–70 years, and not for the younger age groups <45 or 45–59 years. Fatty fish consumption was not associated with a decreased risk of having MetS in any of the age groups. When further adjusting for parity and lactation the statistically significant associations were not longer found (Table 4).

## Discussion

In this large sample from an adult population from Northern Norway, higher fish consumption was associated with a healthier lipid profile with increased HDL-C. A significantly decreased TG was found among men consuming lean fish. Interestingly, only consumption of lean fish, not fatty fish, more than once a week was associated with lower risk of having MetS among those aged 60–70 years.

### Fish consumption and components of metabolic syndrome

#### Lipid levels

In this study fish consumption was associated with healthier blood lipid levels. In the adjusted models, association between consumption of both fatty and lean fish and a decrease in TG were identified. The association did however not remain significant for consumption of fatty fish in men, and consumption of lean fish in women. Further, both consumption of fatty as well as lean fish was associated with an increased HDL-C. However, the association did not remain significant for consumption of lean fish among women when adjusting for age and energy intake.

In other studies fish consumption has been associated with a decreased TG level [6, 9, 13] both among men [6], among women [9]. Both in a prospective cohort study including 3 504 male and female Koreans from the Korean Genome Epidemiology Study [6], in a cross-sectional study from Iran consisting of only women (N = 420) [9], and in an intervention study from Norway [13]. However, a higher level of TG has also been associated with a higher fish consumption in a US population study from the National Heart, Lung, and Blood Institute (NHLBI) Family Heart Study (N = 4 941) [14]. Still, only the intervention study from Norway investigated and found reduced TG after consumption of both fatty (salmon) and lean fish (cod) [13], the other studies [6, 9, 14] did not investigate differences between fatty and lean fish consumption.

In line with previous studies [6, 9, 13, 15], the present study found that fish consumption was associated with an increasing HDL-C. The Korean study found the association among men [6], and the Iranian study found the association among women [9]. The Norwegian study found the association among both men and women, but only significant for fatty fish consumption (P = 0.02) [13].

#### Waist circumference

In this study both fatty and lean fish consumption was associated with a slightly higher WC in the unadjusted models. Interestingly, when adjusting for age and energy intake a higher consumption of lean fish was associated with decreased WC (P = 0.007). However, a higher consumption of fatty fish was associated with a higher WC in men and women.

A reduction in WC has been associated with a higher fish consumption in other studies, both in overweight men consuming fish as part of an energy-restricted diet with lean fish (cod) [16], as well as in a large follow up-study [17]. However, in the latter study they did not find any association between consumption of lean fish and reduction in WC [17]. A Spanish intervention study investigating the effect of fish on CV risk factors in patients with MetS, found that consumption of lean fish (seven servings of Namibian hake per week) was associated with reduced WC [18].

#### Blood pressure

In the present study a significant positive association between blood pressure and fish consumption was observed in the unadjusted models. However, after adjusting for age and energy intake a decrease in blood pressure was found, especially for consumption of lean fish where both SBP and DBP decreased significantly in the whole sample along with an increasing consumption.

Other studies have found associations between high fish consumption and lower blood pressure [9, 18, 19]. Interestingly, both a dietary intervention study from Spain [18] and a dietary intervention study from Iceland [19] found associations between consumption of lean fish and a lower blood pressure. However, the study from Iceland (Cod) [19] found a significant reduction in both SBP (P = 0.001) and DBP (P < 0.001), and the study from Spain (Namibian hake) [18] found a significant reduction only for DBP (P = 0.01). However, not all studies have found associations between fish consumption and lower blood pressure [6].

#### S-glucose

In this study a consumption of both fatty and lean fish was associated with a slightly higher S-glucose, especially among men. However, in this study the S-glucose was not fasting, which may have influenced the result. Further, those with higher fish consumption was older and had a higher energy intake.

Other studies have found that especially omega-3 FAs from fish have been associated with lower blood glucose [20], and have been found to give beneficial effects on the prevention of type 2 diabetes [21].

### Fish consumption and metabolic syndrome

The prevalence of MetS found in this study was 8 %, which is low compared to what others have found in similar age groups [6]. However, data in the present study was collected in 1994/1995, when prevalence of obesity was lower than today [22]. In line with previous studies, an increased prevalence of MetS was found with increasing age [23], with lower prevalence in a young population [9], and higher prevalence in older populations [15].

In this study we found a lower risk of having MetS among participants aged 60–70 years consuming fish once a week or more, compared to those consuming fish less than once a week. Further we found that consumption of lean fish was associated with decreased risk of having MetS. To the best of our knowledge, this association has not been identified in earlier studies. However, this association was not found in the other age groups (<45 and 45–59 years).

Interestingly, in the present study consumption of lean fish was associated with a healthier metabolic profile, than consumption of fatty fish. One possible explanation for the protective effect is that lean fish, such as cod, are considered a superior source of proteins. Proteins in fish have been associated with body weight reduction, through their positive effect on satiety, compared to other animal proteins [24]. The proteins in fish are easily digestible and rich in essential amino acids. Dietary proteins regulate lipid metabolism, depending on the quantity of proteins and composition of the diet, and have been seen to slow absorption and synthesis of lipids, and promote the lipid excretion [25]. Animal studies have suggested that fish protein may have multiple effects on plasma and liver lipids [26], and that consuming fish protein might have beneficial effects on hyperglycaemia and hyperlipidaemia [27, 28]. An improved insulin sensitivity has also been identified in insulin-resistant men and women consuming proteins from cod, compared to other animal proteins [29].

Other studies have found associations between MetS and fish consumption [6–9]. In a follow-up study [6] male and female Koreans (N = 3504) aged 40–69 years without MetS at baseline, associations between fish consumption (sum of dark- or white-meat fish and canned tuna) and incidence of MetS were investigated. In this study MetS was defined according to the adult treatment panel III (ATP III) definition [12], except for WC where alternative criteria were used [30]. They found that the risk of having MetS decreased to less than half (OR 0.43, 95 % CI 0.23–0.83) among men who consumed fish daily, compared with those who consumed fish less than once a week [6]. No significant association was found among women in this study [6].

In a large Finnish population (N = 1334) they found that individuals with MetS had a lower fish consumption (g/d) compared to those without MetS, still only significant among men (P = 0.001) [7]. In this cross-sectional study MetS was defined according to the ATP III definition [31]. After adjusting for age, smoking status, and alcohol consumption an inverse association between fish consumption (10 g/d) and MetS in men was found (OR 0.97, 95 % CI 0.94–1.00). However, after further adjusting for cardiorespiratory fitness (VO_2max_), the association was no longer significant [7], and no significant associations was found in women [7].

An inverse association was also found in a French population, consisting of men aged 45–64 years. In this cross-sectional study they found that the proportions of insulin resistance syndrome (IRS)/MetS, defined according to the ATP III definition [31], decreased along tertiles for fish consumption (adjusted OR 0.57, 95 % CI 0.38–0.86) [8].

The only study investigating associations between fish consumption and MetS in a population of women, is the cross-sectional study from Iran [9]. In this study they identified MetS according to the JIS definition [2], and included female nurses aged >30 years randomly selected from hospitals in Iran (N = 420). In this study they found that a high fish consumption (calculated by the summation of fish and tuna) was inversely associated with prevalence of MetS, where women in the highest tertile of fish consumption were less likely to have MetS compared with those in the lowest tertile (OR 0.35, 95 % CI 0.14–0.88) [9]. Further, this association was strengthened when they adjusted for socio-demography, diet, and BMI (OR 0.04, 95 % CI 0.004–0.61) [9]. However, some studies did not find any associations between fish consumption and MetS prevalence [14, 15]. 

### Gender and age differences

Gender differences have been observed in studies investigating prevalence MetS and dietary patterns containing fish. A Spanish study (N = 808) found an inverse association between following a Mediterranean diet (Med Diet) and risk of having MetS, however only significant in men (P = 0.005) and not in women (P = 0.056) [32]. An inverse association between MetS and a healthy dietary pattern containing fish was also found among women in a Korean study (N = 4984), for highest versus lowest quartile (OR 0.58, 95 % CI 0.50–0.91) [23]. In this study they also stratified by menopausal status, and found that the inverse association only was significant among postmenopausal women (OR 0.60, 95 % CI 0.40–0.86) [23]. Premenopausal woman have higher oestrogen levels, and the oestrogens ability to decrease inflammation and reduce glucocorticoid response may influence the presence of MetS [33].

In women, parity have been associated with higher rates of MetS [34], where a higher number of children increase prevalence of MetS. This is in line with what was found in this study, where women given birth had a higher risk of having MetS (OR 2.69, CI 2.03–3.57), compared to those without children. To our knowledge, no studies have previously adjusted for parity or length of lactation in women [5]. In the present study we found that the associations between consumption of fish and risk of having MetS in the age group 60–70 years disappeared, when adjusting for parity and length of lactation. Women in the age group 60–70 years had on average given birth to twice as many children, still their average total lactation length differed with only 2 months. Gender variations are partly a consequence of differences in sexual hormones levels in men and women, and aging and menopause may lead to differences in lipid profiles [35]. The influence of age on increased plasma TG levels has been reported both in men and women [35]. Further, women have higher HDL-C than men in all ages, and both testosterone levels and estrogen levels have been associated with components of MetS [35].

### Strengths and limitations

The main strength of this study is that it is a large population-based study, with high attendance rate. The study is limited regarding to ethnic diversity, and the vast majority of the participants are Caucasian. As a cross-sectional study, results from this study cannot provide insights on causation between fish consumption and MetS, nevertheless it may provide interesting findings to be further addressed in other studies using different study designs. Dietary data was based on a self-reported FFQ, which might have led to both overestimation and underestimation. However, the reproducibility of dietary data from this self-administered questionnaire was evaluated and a high concordance was found between answers given to the same questions one year apart [36]. This study has no information on the preparation method for fish, and that any positive health effects may diminish or vanish depending on how the meal is prepared. The plasma glucose is non-fasting, and may therefore be less accurate according to the MetS definition.

## Conclusions

In this large sample from an adult population from Northern Norway, higher fish consumption was associated with a healthier lipid profile with increased HDL-C and decreased TG. Fish consumption, particularly lean fish, was associated with a lower risk of having MetS among men aged 60–70 years. The results suggest that fish consumption, particularly lean fish, may have a role in reducing MetS prevalence, and probably related to healthier blood lipid levels. Further investigation is warranted to establish how fish consumption may influence on MetS and its components, particularly regarding differences between fatty and lean fish and age related associations.
